# A Dental Versus a Medical Congress

**Published:** 1888-05

**Authors:** 


A DENTAL VERSUS A MEDICAL CONGRESS.

When the subject of an International Dental Congress was sprung upon the profession a couple of years ago, we urged that the question of the future trend of dentistry be carefully considered and definitely settled. Either we should agree in the drawing yet closer of the bonds which unite us to medicine, or we should sever them altogether. The overwhelming voice of the dentists of America proved to be in favor of a closer union with medicine, and we were committed to the support of a dental section in the Medical Congress. This having been done and our place thus assured us, we should not lightly throw off the obligation until the union with the mother profession has been given a fair trial at least.

The scheme for an International Dental Congress in Paris, in 1889, is necessarily hostile to the dental section of the Medical Congress, which meets in Berlin, in 1890. France will doubtless do what it can to defeat the Berlin meeting, because of the bitter feeling which she entertains toward the Germans—a hatred born of the Franco-Prussian war, and the loss of a part of her territory. Let us be careful how we commit ourselves as partisans for either side. If Paris desires a great dental meeting in 1889, well and good; only do not let us call it a Congress, or take such part in it as shall prove prejudicial to the Medical Congress with which we have cast our lot, and thus perpetuate the unfortunate divisions which marred the Congress of 1887.

